# P-95 - GUIDANCE IN THE WILD: UNDERSTANDING USE AND IMPACT IN SCOTLAND

**DOI:** 10.1093/pubmed/fdag046.096

**Published:** 2026-07-09

**Authors:** Dr Alex Sanchez-Vivar, Mr Colin Sumpter

**Affiliations:** PHS (Public Health Scotland); PHS (Public Health Scotland)

## Abstract

**Background:**

Public Health Scotland (PHS) undertook a national survey in January 2026 to better understand how health protection professionals use, experience, and perceive PHS health protection guidance. The aim was to generate insights to improve guidance quality and usability, strengthen co-production, inform development of a national benchmarking framework, and support continuous improvement in practice.

**Methods:**

A mixed-methods survey (27 items: multiple choice, Likert, and free text) was circulated through professional networks across NHS Boards, Local Authorities, national services, and PHS. Fifty-one responses were received from environmental health officers, consultants in public health, health protection nurses, microbiologists, virologists, healthcare scientists, and care-sector practitioners. Quantitative data were summarised using descriptive statistics, and qualitative free-text responses were analysed thematically to identify emerging themes.

**Results:**

Respondents reported consistently high engagement with PHS guidance, with most using it weekly or monthly. Guidance was widely viewed as useful (53% “extremely useful”; 35% “somewhat useful”) and valued for clarity, readability, structure, accessibility, and consistent formatting. Respondents reported using guidance extensively in case and contact management, outbreak investigation, IMT/PAG decision-making, operational procedures, and laboratory workflows. Many described tangible practice changes and viewed guidance as supporting more consistent, evidence-based decisions across organisations.

Areas for improvement included website navigation, PDF usability, timeliness of updates, clarity of definitions, distinguishing mandatory from advisory actions, and making Scottish-specific recommendations more visible. While views on co-production and transparency were largely positive, respondents identified gaps in stakeholder representation (e.g., pharmacy, occupational health, laboratory managers, rural/remote services) and uncertainty about how equity considerations are embedded. Participants also suggested future guidance topics and highlighted the need to update older documents and reduce duplication with UKHSA.

**Conclusions:**

PHS health protection guidance is widely trusted, frequently used, and central to operational practice in Scotland. Enhancing usability, update processes, clarity, stakeholder involvement, alignment with UKHSA, and visibility of equity considerations would further strengthen its impact and support consistent implementation across settings.

